# Effects of Different Types of Front-of-Pack Labelling Information on the Healthiness of Food Purchases—A Randomised Controlled Trial

**DOI:** 10.3390/nu9121284

**Published:** 2017-11-24

**Authors:** Bruce Neal, Michelle Crino, Elizabeth Dunford, Annie Gao, Rohan Greenland, Nicole Li, Judith Ngai, Cliona Ni Mhurchu, Simone Pettigrew, Gary Sacks, Jacqui Webster, Jason H. Y. Wu

**Affiliations:** 1 The George Institute for Global Health, University of New South Wales, Sydney, NSW 2042, Australia; mcrino@georgeinstitute.org.au (M.C.); edunford@georgeinstitute.org.au (E.D.); agao@georgeinstitute.org.au (A.G.); nli@georgeinstitute.org.au (N.L.); jwebster@georgeinstitute.org.au (J.W.); jwu1@georgeinstitute.org.au (J.H.Y.W.); 2 The Charles Perkins Centre, University of Sydney, Sydney, NSW 2006, Australia; 3 Royal Prince Alfred Hospital, University of New South Wales, Sydney, NSW 2050, Australia; 4 Department of Epidemiology and Biostatistics, Imperial College London, London SW7 2AZ, UK; 5 Carolina Population Center, The University of North Carolina at Chapel Hill, Chapel Hill, NC 27516, USA; 6 National Heart Foundation of Australia, Melbourne, VIC 3000, Australia; Rohan.Greenland@heartfoundation.org.au; 7 Bupa, Brisbane, QLD 4001, Australia; judith.ngai@bupa.com.au; 8 National Institute for Health Innovation, University of Auckland, Auckland 1010, New Zealand; c.nimhurchu@auckland.ac.nz; 9 School of Psychology and Speech Pathology, Curtin University, Bentley, WA 6102, Australia; simone.pettigrew@curtin.edu.au; 10 School of Health and Social Development, Deakin University, Melbourne, VIC 3008, Australia; gary.sacks@deakin.edu.au

**Keywords:** food labelling, food industry, food purchases, policy, randomised trial

## Abstract

Background: Front-of-pack nutrition labelling may support healthier packaged food purchases. Australia has adopted a novel Health Star Rating (HSR) system, but the legitimacy of this choice is unknown. Objective: To define the effects of different formats of front-of-pack labelling on the healthiness of food purchases and consumer perceptions. Design: Individuals were assigned at random to access one of four different formats of nutrition labelling—HSR, multiple traffic light labels (MTL), daily intake guides (DIG), recommendations/warnings (WARN)—or control (the nutrition information panel, NIP). Participants accessed nutrition information by using a smartphone application to scan the bar-codes of packaged foods, while shopping. The primary outcome was healthiness defined by the mean transformed nutrient profile score of packaged foods that were purchased over four weeks. Results: The 1578 participants, mean age 38 years, 84% female recorded purchases of 148,727 evaluable food items. The mean healthiness of the purchases in the HSR group was non-inferior to MTL, DIG, or WARN (all *p* < 0.001 at 2% non-inferiority margin). When compared to the NIP control, there was no difference in the mean healthiness of purchases for HSR, MTL, or DIG (all *p* > 0.07), but WARN resulted in healthier packaged food purchases (mean difference 0.87; 95% confidence interval 0.03 to 1.72; *p* = 0.04). HSR was perceived by participants as more useful than DIG, and easier to understand than MTL or DIG (all *p* < 0.05). Participants also reported the HSR to be easier to understand, and the HSR and MTL to be more useful, than NIP (all *p* < 0.03). Conclusions: These real-world data align with experimental findings and provide support for the policy choice of HSR. Recommendation/warning labels warrant further exploration, as they may be a stronger driver of healthy food purchases.

## 1. Introduction

Poor diet due to inadequate intake of healthy foods and excess intake of unhealthy/junk foods—resulting in excess consumption of adverse nutrients and excess energy—is a leading preventable risk factor for poor health in the world [1]. Improving diets was identified as a priority for global action at the United Nations High-Level Meeting on Non-Communicable Diseases (NCDs) in 2011 [2] and in the resulting World Health Organization Global NCD Action Plan [1].

Nutrition labelling is a policy tool that can be used to promote healthy food choices and better eating habits. Nutrition labelling may also drive product reformulation and enhance the average quality of food purchases [3,4,5]. A basic nutrient declaration in the form of a back-of-pack nutrient declaration is required on pre-packaged foods in Australia and many other countries [6], but the format of presentation is often difficult for consumers to understand and act upon [7]. Front-of-pack interpretive nutrition labels that use graphics and colours to depict nutrient content are likely to be a better option for consumers because they provide information in an easily understandable format.

The multiple traffic-light (MTL) label, which signposts levels of fats, sugars, and salt using icons coloured red (high), amber (medium), or green (low) is the best known example of an interpretive front-of-pack label [8]. In 2014, the Australian Government adopted the ‘Health Star Rating (HSR)’ front-of-pack labelling scheme [9], which has similarities to the retailer-developed ‘Guiding Stars’ programme in the United States (US) [10]. The HSR scheme uses a nutrient profiling algorithm to assign between 0.5 (least healthy) and 5.0 (most healthy) stars to a food in half star increments. The ‘Daily Intake Guide’ (DIG) is the non-interpretive front-of-pack labelling system that is partially implemented by the Australian food industry [11], but recent research suggests that it is not especially helpful to consumers [12]. Other nations have adopted systems based upon cautioning against the least healthy options using warning labels [13], which may be particularly effective because of the very explicit nature of the messaging provided.

Multiple experimental studies [12,14,15,16,17,18,19,20] have described the comparative effects of different labelling systems on consumer preferences and the ability to correctly identify healthy and unhealthy food items. However, there are relatively few data to define effects of food labels on food purchasing behaviour in the real world and the findings are mixed [10,12,21,22,23]. Consequently, the implementation of front-of-pack labelling in most countries has been limited. Using our novel FoodSwitch smartphone technology [24], we sought to test the validity of the Australian Government’s decision to adopt HSR labelling. We did this by providing consumers with different formats of front-of-pack labelling in the form of point-of-sale information to aid healthy food purchases. Our large-scale randomised trial compared the effects of HSR against three other types of nutrition labels (TLL, DIG, and recommendations/warnings), and also against control (Nutrient Information Panel, NIP) to determine the effects on the healthiness of food purchases.

## 2. Participants and Methods 

This was a randomized, double-blind, placebo-controlled, parallel-group trial. The entire study was done through a smartphone application with no direct contact between researchers and participants. Recruitment commenced in September 2014 and follow-up was completed in May 2016.

### 2.1. Participants

Participants (1) were 18 years and older adult residents in Australia; (2) owned an iPhone or Android smartphone; (3) shopped at a supermarket at least once a week; (4) were the regular main shopper for the household; (5) were available for a continuous 5-week period; (6) were able to read and understand English; and, (7) consented to take part in the trial. Exclusion was based upon another member of the same household having already been enrolled in the study or failure to successfully complete the run-in phase of the study.

### 2.2. Recruitment

Participants were recruited across Australia through adverts in local newspapers, social media, mail drops, direct contact at supermarkets and community venues, research team networks, radio, and via an online consumer panel. Eligible participants were directed to download the “Food Label Trial” smartphone application, through which informed consent was obtained and eligibility determined. Baseline data collection comprised about 25 questions documenting basic socio-demographic variables and understanding about nutrition and food labels.

### 2.3. Run-in

Consenting, potentially eligible participants entered the run-in phase of the trial. During this period, they recorded their food and beverage purchases for one week using the smartphone application to scan the barcodes of purchased products and photograph the corresponding till receipts using the smartphone camera. After one week, the potential participants who had scanned at least 15 purchased barcoded grocery items were randomised into the study. Failure to submit 15 or more grocery items resulted in ineligibility.

### 2.4. Randomisation

Randomisation was done by an algorithm built into the smartphone application using randomisation codes that were prepared by a statistician based at the George Institute for Global Health. Randomisation was in a 1:1:1:1:1 ratio without stratification.

### 2.5. Intervention and Control

**Figure 1:**
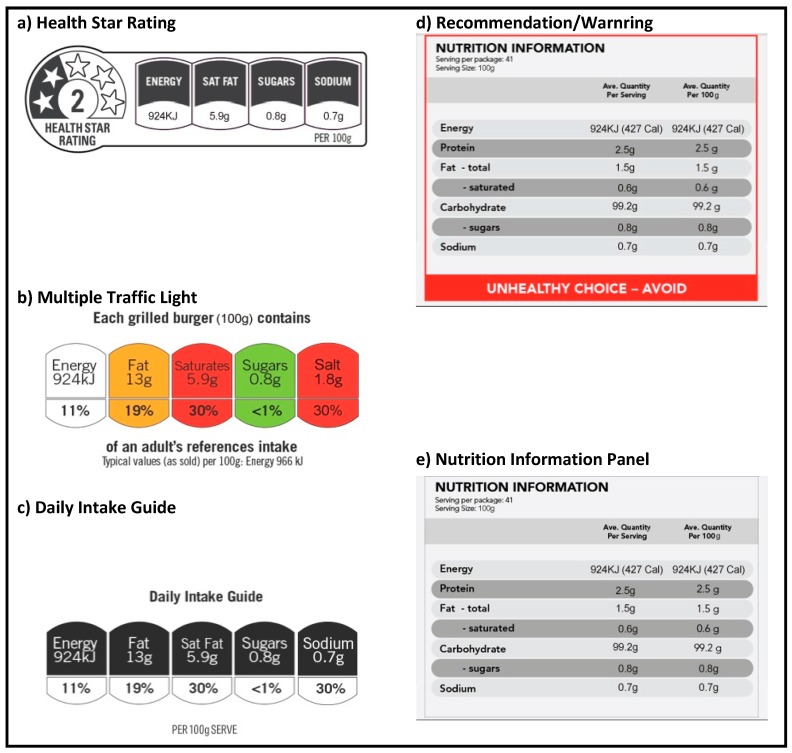
Label formats investigated.

Immediately after randomisation, the functionality of the smartphone application on the participant’s phone was updated such that it gave the user access to their randomly assigned modality of food labelling: HSR; MTL; DIG; recommendations/warnings (WARN); or, NIP (Figure 1). During the subsequent four-week period, all of the participants were encouraged to use the smartphone application every time that they purchased packaged food at any retail outlet. To use the labelling functionality of the application, participants moved the smartphone camera over the barcode of a product. The camera acquired the barcode image and immediately provided onscreen information about product healthiness formatted according to the label group to which the participant was randomised. A random sample of similar foods was also shown on-screen with the same label format applied to allow users to see if there were healthier alternative products available that they might choose instead.

### 2.6. Data Collection and Follow-Up

For the duration of the four-week intervention period, intervention and control group participants were asked to record all of the packaged food purchases that were made by scanning the barcodes and capturing images of till receipts using the smartphone camera. The data and images were transferred to a central online database using functionality built into the app. In addition, participants were asked to keep the hard copies of all their till receipts that were mailed in using reply paid envelopes. At the completion of the intervention period, participants were asked to provide information about their use of the smartphone application during the preceding month and record their perceptions of the form of food labelling to which they were assigned.

### 2.7. Outcomes

The primary outcome was the mean transformed nutrient profile score (NPS) for all food and beverage products that were purchased over the four-week intervention period. The nutrient profile score was calculated using the updated Food Standards Australia New Zealand nutrient profiling calculator and food composition data obtained from the Australian FoodSwitch database [24]. Transformation was done because the nutrient profile score differs for products that fall into each of three defined food categories (i.e., from −13 to 40 for Category 1 comprising beverages, from −18 to 40 for Category 2 comprising selected dairy and fats, and from −18 to 81 for Category 3 comprising other food types). The transformation standardises the NPS to a 0–100 range for every product using the equation transformed NPS = (NPS—Category score for the least healthy product)/range of Category score ×100. Secondary outcomes were the mean saturated fat per 100 g, the mean total sugar per 100 g, the mean sodium per 100 g, the mean energy content per 100 g, and the mean food expenditure in Australian dollars for all food and beverage products purchased over the four-week intervention period. Self-reported preference and utility of the assigned labelling system was also collected.

### 2.8. Ethics

The study received approval from the University of Sydney Ethics Committee, was done in accordance with the principles of the Declaration of Helsinki and was registered at the Australian New Zealand Clinical Trials Registry (ACTRN12614000964617).

### 2.9. Statistics

**Statistical power**—The planned sample size was 2500 randomised individuals that were assigned in a 1:1:1:1:1 ratio to one of the four intervention groups or control with the primary goal of achieving 90% power (at alpha = 0.05) to detect a 2 unit or greater difference in the mean transformed nutrient profile of purchased foods (healthiness score derived from the nutrient profile system) between each specified pair of groups. Following the adoption of the HSR system by the Australian government shortly after trial commencement, the primary objective of the study was modified to test whether this decision was reasonable by testing whether there was any evidence that the HSR system was inferior to the alternative possible choices (MTL, DIG, or warnings), with superiority of each form of labelling against NIP control being tested as secondary objectives. The achieved sample size of 1578 provided more than 80% power (at alpha = 0.05) to demonstrate non-inferiority with a margin of 2 units (2%) for the primary outcome. Non-inferiority margins for the secondary outcomes were set at 10% of the mean value for each.

**Analysis**—Baseline characteristics are presented as means with standard deviation (SD) for continuous variables and as percentages for categorical variables. The primary efficacy analyses used a mixed model that accommodated the clustering of food items that were purchased by an individual to estimate the mean differences and 95% confidence intervals (95% CI) for the transformed nutrient profile score between randomised groups. The mean transformed nutrient profile score for products that were purchased during the run-in period was included as a covariate in the model. Statistical testing for primary, secondary, and process outcomes was done at the 5% significance level, with no formal adjustment for multiplicity. All of the evaluations were performed on the principle of intention to treat using all of the randomized participants with available data. For the process and preference outcomes, we used analysis of variance to test the differences in average responses between all of the randomised groups, and unpaired *t*-test to contrast specific pairs of randomised groups. The constancy of effects was explored across subgroups that were defined by age, gender, education, prior use of the FoodSwitch smartphone application [24], healthiness of baseline diet, interest in healthiness of diet, income, use of nutrition labels, nutrition knowledge, and number of items purchased during run-in by fitting interaction terms to the relevant models. The primary comparisons made were testing of non-inferiority of HSR vs. MTL, HSR vs. DIG, and HSR vs. WARN, followed by testing of superiority for each comparison where non-inferiority was met. Secondary superiority comparisons were then made for HSR vs. NIP, MTL vs. NIP, DIG vs. NIP, and WARN vs. NIP. To place the trial in context, we also did a fixed effects meta-analysis incorporating the data from this trial and a recently reported sister trial done in New Zealand [25] that tested two directly comparable comparisons using the same methods.

## 3. Results

**Figure 2:**
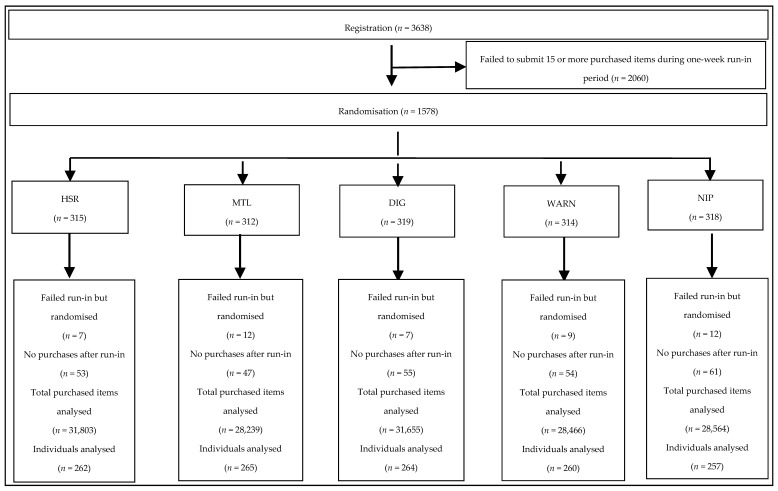
CONSORT diagram. Health Star Rating (HSR), Multiple Traffic Lights (MTL), Daily Intake Guide (DIG), Recommendations/warnings (WARN), and Nutrition Information Panel (NIP).

There were 3638 individuals who provided informed consent and entered the run-in phase of the study. Of these, 2060 (57%) did not submit data on 15 or more purchased food items within seven days and were therefore excluded from further participation (Figure 2). The 1578 randomised individuals were 83.8% female, had a mean age of 37.9 years, had completed tertiary education in 77.5% of cases, and mostly came from households with a combined average income of >A$50,000. There were 47 individuals who did not meet the run-in criteria who were nonetheless randomised due to a technical error and were included in the primary analyses under the principle of intention to treat. The characteristics of the five randomised groups were well balanced for all of the measured characteristics, including self-reported measures of the healthiness of current diet, interest in healthy eating, knowledge about nutrition, and prior use of food labels (Table 1).

### 3.1. Use of the Smartphone Application and Provision of Follow-Up Data

On average, during run-in, the 1578 randomised participants provided data on a mean of 32 (SD 55) items, purchased during a mean of 3.0 (SD 2.8) episodes of shopping with a mean transformed nutrient profile of score of 59.7 (SD 17.4) that was not different between randomised groups (*p* = 0.64). During follow-up, there was a mean of 79 (SD 82) additional items recorded for each individual at a mean of 7.8 (SD 7.1) shopping episodes. On average, participants scanned a mean of 31 (SD 56) food barcodes during the randomised intervention period to see front-of-pack labelling information. There were no differences in any of these metrics between randomised groups (all *p* ≥ 0.46). There were 1163/1578 individuals (220 to 246 per randomised group) who provided information at trial completion about their perceptions of the labelling system to which they were assigned.

### 3.2. Healthiness of Food Purchases

The HSR system was non-inferior to the other three front-of-pack labelling systems in terms of the primary outcome and the mean transformed nutrient profile score (all *p*-values for non-inferiority <0.001) (Table 2). There was, however, no evidence that the HSR system was superior to the alternatives for this outcome. The HSR was also non-inferior for all secondary outcomes in all of the comparisons (all *p*-values for non-inferiority ≤0.05) except sodium (all *p* non-inferiority ≥ 0.08).

For the primary outcome of mean transformed nutrient profile score, the comparisons of each front-of-pack labelling system against NIP control showed superiority for WARN (*p* = 0.04), but no effect for HSR, MTL, or DIG (all *p* ≥ 0.09) (Table 3). For the secondary outcomes, MTL resulted in reduced purchases of sugar (*p* = 0.04) and WARN in a higher average expenditure on purchased products (*p* = 0.05). There were no statistically significant effects that were identified for other outcomes (all *p >* 0.07).

There was no evidence of effect modification for any of the pre-specified subgroup analyses for the primary outcome, except for self-reported nutrition knowledge at baseline (*p* interaction = 0.001), where HSR and MTL appeared to perform more consistently across higher and lower levels of self-reported nutrition knowledge when compared to DIG, WARN, and NIP.

### 3.3. Perceptions of the Labelling Systems

The HSR label format was non-inferior to the other three front-of-pack labelling systems in terms of participant perceptions about the usefulness of the labels, the comprehensibility of the labels, the desire to see the labels on all of the products, and the impact on user nutrition knowledge (all *p* non-inferiority < 0.001). Furthermore, the HSR label format was statistically significantly better than other formats in seven out of twelve comparisons (all *p <* 0.05), with eleven out of twelve point estimates of effect for these outcomes favouring the HSR format over the alternatives in the pairwise comparisons (Table 4).

## 4. Discussion

These data provide endorsement of the Australian Government’s decision to adopt the HSR as their recommended front-of-pack labelling system. The HSR was as good as any other label that was tested in terms of the healthiness of purchased foods, while being superior to others in several aspects of consumer preference. It was also a front runner in terms of its utility across groups with a range of different levels of nutritional knowledge. This is an important attribute given the greater burden of diet-related ill health amongst less educated sectors of the population and the known difficulties that some groups have in understating nutrition information.

### 4.1. Strengths and Weaknesses of the Trial

The trial benefitted from its large size, randomised design, robust measure of the healthiness of food purchases, and the real-world setting in which it was conducted. Substantial efforts were under-taken to minimise the risk of reporting biases, with participants being informed only that they were participating in a study investigating knowledge and effects of food labels on their purchases, but without knowledge of the details of the interventions that were under investigation. To encourage standardized reporting across randomized groups, participants were advised that those who returned post-randomisation shopping data would be provided with A$100 in online shopping vouchers in acknowledgement of their time spent completing study procedures. Study staff entering the data were masked to the randomised allocation of the participant from which the data derived.

The use of a smartphone app to provide point-of-sale information and collect outcome data was innovative. At the same time, however, a key challenge to our approach was the imperfect replication of on-pack labels by the smartphone-based approach. While universal printed on-pack labels would be immediately apparent, trial participants were required to open and use the smartphone application to see front-of-pack labelling information, and to scan multiple products each time that they went shopping to make comparisons. The metrics on app usage indicate that scanning was done for only a subset of food purchases and it is likely that this has resulted in the underestimation of the impact that might be achieved with universal front-of-pack printed labels. Even if consumers did not actively use printed front-of-pack labels for every purchase, it is possible that universal printed front-of-pack labelling would subconsciously influence many purchases.

The large proportion of participants self-reporting high levels of nutritional knowledge at baseline, the high household incomes and the substantial proportion with tertiary education indicate that the trial population is not representative of Australian shoppers. Power to investigate for interactions between these characteristics and the effects of the intervention was limited, and there remains some uncertainty about the generalisability of the findings. It is also of note that we provided no training on the use of any format of front-of-pack labelling and it is possible that findings might be modified if there was concurrent community education. The DIG format has been present on the packs of some Australian foods for many years and the HSR format was present on a small subset of foods when the study was conducted. It is possible that this could have differentially influenced the capacity of consumers to use the various label formats that were tested in the study.

### 4.2. The Findings in Context

**Figure 3:**
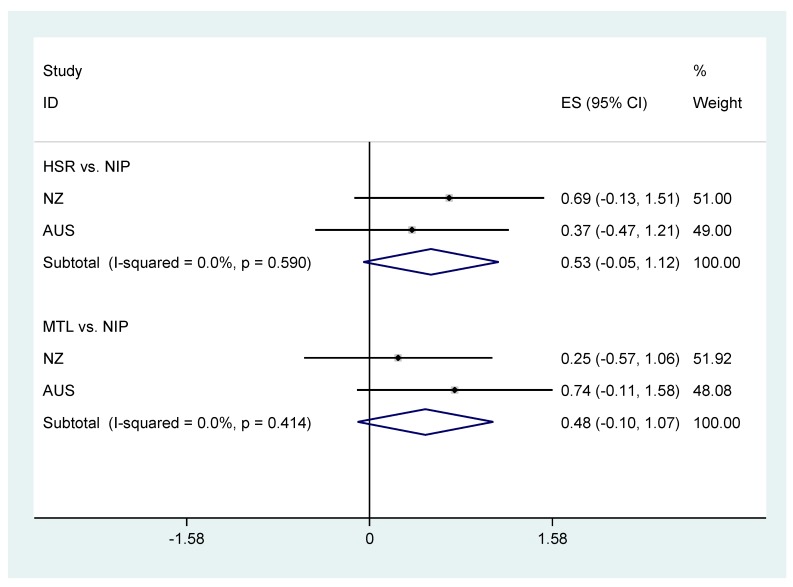
Fixed effects meta-analysis of the effects of health star ratings and multiple traffic lights compared to control on healthiness of food purchases in the current trial and a sister trial done in New Zealand (mean differences and 95% confidence interval). ES = Effect size; 95% CI = 95% confidence interval; HSR = Health Star Rating; MTL = Multiple Traffic Light; NIP = Nutrition Information Panel; NZ = New Zealand; AUS = Australia. I-squared statistic indicates between trial differences in contributing trial results beyond chance and *p*-value is result for test of heterogeneity of trial results.

There are few comparably robust data describing the effects of different types of front-of-pack labelling on consumer behaviour, with most prior research done in experimental settings or using non-randomised designs [15,16]. The findings of this trial are aligned with the existing experimental evidence base relating to the HSR, and front-of-pack labelling more broadly, and in particular with the data from the recently reported sister trial to this project done in New Zealand [25]. That trial was also unable to show a clear superiority of interpretive front-of-pack labelling when compared to control in terms of the healthiness of food choices, but point estimates of effect for the healthiness of food purchases were favourable and HSR and MTL performed better in terms of consumer preference. The meta-analysis of the comparisons common across the two studies (HSR vs. NIP and MTL vs. NIP) showed comparable directions and sizes of effects to our study, and the summary data from the trials suggest that both HSR and MTL are likely to result in healthier food purchases, although both fell just short of standard statistical significance (Figure 3).

The borderline statistically significant healthier food purchases achieved with WARN when compared to NIP is likely to reflect the strength of the advice that was provided by the WARN label format. It is widely accepted in the tobacco control field that explicit on-pack warnings change purchasing behaviour [26], and it is likely that more extreme labelling of foods, such as the salt warnings used in Finland, could produce changes in food choices [27]. The warnings/recommendations we used in the WARN arm of the trial were moderate by comparison to the messaging used on tobacco products, but they were much more direct than the HSR, MTL, or DIG formats. We included the WARN arm in the study because we wished to robustly test the question of whether front-of-pack labelling delivered in a plausible, but maximally robust format, could change behaviour. While the WARN format was not rated as highly by consumers, if the effect on the healthiness of food purchases was confirmed as real, this would provide a compelling case for the adoption of this form of labelling. The WARN arm of the trial is the closest approximation to the warning labels that have been adopted in several overseas jurisdictions [13].

## 5. Conclusions and Implications

In conclusion, we used a trial design with a primary non-inferiority objective and showed with confidence that the selection of HSR was an appropriate policy decision for Australia in the context of strong industry opposition to the MTL and evidence suggesting DIG to be inferior [3,28,29,30]. While the HSR was not shown to result in healthier food purchases, it was clearly preferred by consumers, with comparable findings in the parallel New Zealand study. For countries without a front-of-pack labelling system, our data suggest that the HSR is an appropriate choice, but a case could also be made for WARN or MTL, or for the integration of elements of the WARN and MTL formats into an updated HSR system. There appears, however, to be little rationale to support the DIG format (also described as guideline daily amount), which was the industry-preferred option until the adoption of HSR.

## Figures and Tables

**Table 1:** Baseline characteristics.

	*n*	MTL (312)	DIG (319)	HSR (315)	WARN (314)	NIP (318)	All (1578)
Age, years (mean ± SD)	1575	38.5 ± 11.5	38.2 ± 10.9	37.7 ± 11.2	36.9 ± 11.3	38.2 ± 11.1	37.9 ± 11.2
Female (%)	1578	263 (84.3)	275 (86.2)	262 (83.2)	265 (84.4)	257 (80.8)	1322 (83.8)
Household income (A$) (*n*, %)	1431						
<$50,000		45 (14.4)	59 (18.5)	51 (16.2)	50 (15.9)	53 (16.7)	258 (16.3)
$50,000–$100,000		119 (38.1)	99 (31.0)	113 (35.9)	110 (35.0)	107 (33.6)	548 (34.7)
>$100,000		123 (39.4)	127 (39.8)	119 (37.8)	125 (39.8)	131 (41.2)	625 (39.6)
Highest level of education (*n*, %)	1578						
Primary/Secondary		57 (18.3)	78 (24.5)	81 (25.7)	64 (20.4)	64 (20.1)	344 (21.8)
Tertiary		167 (53.5)	149 (46.7)	148 (47.0)	160 (51.0)	167 (52.5)	791 (50.1)
Post-graduate		86 (27.6)	90 (28.2)	83 (26.3)	89 (28.3)	84 (26.4)	432 (27.4)
None of the above		2 (0.6)	2 (0.6)	3 (1.0)	1 (0.3)	3 (0.9)	11 (0.7)
Employment status (*n*, %)	1578						
Full Time		133 (42.6)	128 (40.1)	131 (41.6)	134 (42.7)	130 (40.9)	656 (41.6)
Part Time		89 (28.5)	88 (27.6)	87 (27.6)	92 (29.3)	86 (27.0)	442 (28.0)
Other/Unemployed		90 (28.8)	103 (32.3)	97 (30.8)	88 (28.0)	102 (32.1)	480 (30.4)
Prior use of FoodSwitch (*n*, %)	1578	24 (7.6)	28 (8.8)	21 (6.7)	20 (6.4)	23 (7.2)	116 (7.4)
Number in household (mean ± SD)	1578	3.2 ± 1.4	3.1 ± 1.4	3.2 ± 1.4	3.0 ± 1.3	3.2 ± 1.4	3.2 ± 1.4
Number in household under 18 (mean ± SD)	1529	1.0 ± 1.1	1.1 ± 1.2	1.0 ± 1.2	0.9 ± 1.1	1.1 ± 1.2	1.0 ± 1.2

SD = standard deviation; 147 participants declined to answer question regarding annual income. Health Star Rating (HSR), Multiple Traffic Lights (MTL), Daily Intake Guide (DIG), Recommendations/warnings (WARN), and Nutrition Information Panel (NIP).

**Table 2:** Effects of health star ratings compared to other types of front-of-pack labelling on healthiness of food purchases (mean differences and 95% confidence interval).

	HSR vs. MTL	*p* Non-Inferiority *p* Superiority	HSR vs. DIG	*p* Non-Inferiority *p* Superiority	HSR vs. WARN	*p* Non-Inferiority *p* Superiority
**Primary outcome**						
Mean transformed nutrient profile score	−0.37 (−1.20, 0.46)	<0.001 0.38	0.68 (−0.14, 1.50)	<0.001 0.10	−0.51 (−1.33, 0.32)	<0.001 0.23
**Secondary outcomes**						
Mean total sugar g/100 g	0.41 (−0.43, 1.25)	0.05 0.34	−0.12 (−0.95, 0.71)	0.002 0.77	0.10 (−0.74, 0.94)	0.01 0.82
Mean sodium mg/100 g	13 (−16, 41)	0.09 0.39	23 (−5, 51)	0.25 0.11	12 (−16, 41)	0.08 0.39
Mean saturated g/100 g	0.11 (−0.27, 0.48)	0.02 0.58	−0.21 (−0.58, 0.15)	<0.001 0.26	−0.05 (−0.42, 0.32)	0.001 0.80
Mean energy content kJ/100 g	25 (−10, 61)	<0.001 0.17	−7 (−43, 28)	<0.001 0.68	8 (−28, 44)	<0.001 0.66
Mean spend/4 weeks (A$)	0.03 (−0.12, 0.19)	<0.001 0.67	0.06 (−0.09, 0.22)	<0.001 0.45	−0.02 (−0.18, 0.14)	<0.001 0.83

Non-inferiority: 1-sided *p*-value testing at *p <* 0.05. Superiority: 2-sided *p*-value testing at *p <* 0.05. Non-inferiority margin for primary outcome was 2 units on 100 unit scale and 10% of mean for each value for secondary outcomes. Health Star Rating (HSR), Multiple Traffic Lights (MTL), Daily Intake Guide (DIG), Recommendations/warnings (WARN), Nutrition Information Panel (NIP).

**Table 3:** Effects of each type of front-of-pack labelling compared to control on healthiness of food purchases (mean differences and 95% confidence interval).

	MTL vs. NIP	*p* Superiority	DIG vs. NIP	*p* Superiority	HSR vs. NIP	*p* Superiority	WARN vs. NIP	*p* Superiority
**Primary outcome**								
Mean transformed Nutrient Profile Score	0.74 (−0.11, 1.58)	0.09	−0.31 (−1.15, 0.52)	0.46	0.37 (−0.47, 1.21)	0.39	0.87 (0.03, 1.72)	0.04
**Secondary outcomes**								
Mean total sugar g/100 g	−0.89 (−1.74, −0.03)	0.04	−0.35 (−1.20, 0.49)	0.41	−0.48 (−1.33, 0.37)	0.27	−0.57 (−1.43, 0.28)	0.19
Mean sodium mg/100 g	−2 (−31, 28)	0.91	−12 (−41, 17)	0.41	11 (−18, 40)	0.47	−2 (−31, 28)	0.91
Mean saturated fat g/100 g	−0.30 (−0.68, 0.08)	0.12	0.02 (−0.35, 0.39)	0.92	−0.19 (−0.57, 0.18)	0.31	−0.14 (−0.52, 0.23)	0.45
Mean energy content kJ/100 g	−26 (−63, 11)	0.16	7 (−29, 43)	0.71	−1 (−37, 36)	0.98	−8 (−45, 28)	0.65
Mean spend/4 weeks (A$)	0.11 (−0.05, 0.27)	0.17	0.08 (−0.07, 0.24)	0.29	0.14 (−0.01, 0.30)	0.07	0.16 (0.002, 0.32)	0.05

Superiority: 2-sided *p*-value testing at *p <* 0.05. Health Star Rating (HSR), Multiple Traffic Lights (MTL), Daily Intake Guide (DIG), Recommendations/warnings (WARN), Nutrition Information Panel (NIP).

**Table 4:** Perceptions of health star ratings compared to other types of front-of-pack labelling (mean differences and 95% confidence interval).

	HSR vs. MTL	*p* Non-Inferiority *p* Superiority	HSR vs. DIG	*p* Non-Inferiority *p* Superiority	HSR vs. WARN	*p* Non-Inferiority *p* Superiority
Current nutrition knowledge after using the study app	0.36 (−0.03, 0.75)	<0.001 0.07	0.44 (0.06, 0.82)	<0.001 0.02	0.25 (−0.13, 0.64)	<0.001 0.20
How useful were the nutrition labels shown in the app	−0.01 (−0.52, 0.50)	<0.001 0.97	0.76 (0.26, 1.26)	<0.001 0.003	0.07 (−0.43, 0.58)	<0.001 0.78
How easy to understand were the labels shown in the app	0.62 (0.19, 1.05)	<0.001 0.005	1.02 (0.60, 1.44)	<0.001 <0.001	0.22 (−0.21, 0.64)	<0.001 0.32
How useful would it be to have those labels printed on every food package	0.44 (−0.001, 0.88)	<0.001 0.05	0.70 (0.27, 1.13)	<0.001 0.002	0.43 (−0.003, 0.87)	<0.001 0.05

Non-inferiority: 1-sided *p*-value testing at *p <* 0.05. Superiority: 2-sided *p*-value testing at *p <* 0.05. Non-inferiority margin was 1 unit on 10 unit scale for all outcomes. Health Star Rating (HSR), Multiple Traffic Lights (MTL), Daily Intake Guide (DIG), Recommendations/warnings (WARN), Nutrition Information Panel (NIP).

## Abbreviations

CI	Confidence Interval
DIG	Daily Intake Guides
HSR	Health Star Rating
MTL	Multiple Traffic Light Labels
NIP	Nutrition Information Panel
WARN	Recommendations/Warnings
